# Articulated Arm Coordinate Measuring Machine Calibration by Laser Tracker Multilateration

**DOI:** 10.1155/2014/681853

**Published:** 2014-01-29

**Authors:** Jorge Santolaria, Ana C. Majarena, David Samper, Agustín Brau, Jesús Velázquez

**Affiliations:** Departamento de Ingeniería de Diseño y Fabricación, Edificio Torres Quevedo, EINA, Universidad de Zaragoza, 50018 Zaragoza, Spain

## Abstract

A new procedure for the calibration of an articulated arm coordinate measuring machine (AACMM) is presented in this paper. First, a self-calibration algorithm of four laser trackers (LTs) is developed. The spatial localization of a retroreflector target, placed in different positions within the workspace, is determined by means of a geometric multilateration system constructed from the four LTs. Next, a nonlinear optimization algorithm for the identification procedure of the AACMM is explained. An objective function based on Euclidean distances and standard deviations is developed. This function is obtained from the captured nominal data (given by the LTs used as a gauge instrument) and the data obtained by the AACMM and compares the measured and calculated coordinates of the target to obtain the identified model parameters that minimize this difference. Finally, results show that the procedure presented, using the measurements of the LTs as a gauge instrument, is very effective by improving the AACMM precision.

## 1. Introduction 

In recent years, there has been an increasing interest in AACMM's because of their advantages in terms of accuracy, portability and suitability for inspection and quality control tasks in machining tool processes and in the automotive and aerospace industry [1].

Nevertheless, few researches have focused on the calibration of these mechanisms. Moreover, there is an absence of standards on verification and calibration procedures. For that reason, AACMM manufacturers have developed its own evaluation procedures. These evaluation methods are based on the three main standards for performance evaluation in current CMM's, UNE-EN ISO 10360, ASME B89.4.1, and VDI/VDE 2617 and are still carried out today to compare and evaluate the accuracy of an arm from the point of view of the CMM's. In [1], the author presented a procedure to check the performance of coordinate measuring arms by calculating the distances between the centers of different spheres. The results obtained were compared with the application of the ANSI/ASME B89 volumetric performance test showing good agreement between the two approaches and a cost reduction. In [2], Shimojima et al. presented a new method to estimate the uncertainty of a measuring arm using a tridimensional gauge. This method consists of a fat plate with 9 spheres fixed at three different heights with respect to the metallic surface of the plate. Then the spheres centers are measured with the measuring arm at different locations and orientations, and distances between spheres centers are compared to the nominal distances to evaluate the measuring performance of the arm. Other works have been found in the literature whose main goal is also to evaluate the performance of measuring arms [3–5].

However, the AACMM presents different characteristics, and different verification procedures are therefore required. A point clearly defines a position of the three machine axes for a CMM. Nevertheless, the possible positions of the AACMM elements to achieve a fixed point defined in the measurement volume are practically infinite. Moreover, for CMMs, evaluation tests can be performed to extract the positioning errors, allowing correction models to be implemented [1]. Thus, a high level of maintenance of the physical-mathematical relations between the error model parameters and the error physically committed by the machine can be achieved. However, the application of these models does not make sense in AACMM's, given the difficulty of directly relating the error committed with the model parameters, which are obtained by using optimization procedures.

The calibration procedure consists of identifying the geometric parameters in order to improve the model accuracy. This procedure allows us to obtain correction models to establish corrections in the measurements results and to quantify the effects of the influence variables in the final measurement. To achieve this goal, the following five steps are usually carried out: determination of the kinematic model by means of nonlinear equations, data acquisition, optimization or geometric parameter identification, model evaluation, and, finally, identification of the error sources and implementation of correction models.

The first step, determination of the kinematic model, consists of obtaining the non-linear equations that relate the joint variables to the position and orientation of the end-effector and the initial values of nominal geometric parameters. One of the most widely used geometric methods for modelling a mechanism is the well-known Denavit-Hartenberg method [6], which models the joints with four parameters. One of the limitations of this method appears in those mechanisms that present two consecutive parallel joint axes. In this case, an infinite number of common normals of the same length exist, and the location of the axis coordinate system may be defined arbitrarily. Some studies [7–10] present methods to obtain a complete, equivalent, and proportional model.

The number of parameters is fixed when the kinematic model is determined, and this value will depend on the selected method. Moreover, there are a maximum number of parameters that must be identified, and the model accuracy does not have to improve by adding extra parameters [11]. In this work, the author determined that four parameters must be considered for each revolute joint, two of which must be orientational, and for a prismatic joint two orientational parameters are necessary, applied about the noncollinear axes before and perpendicular to the translational joint axes. Nongeometric errors are usually compensated by adding parameters in the geometric model [12].

The second step is data acquisition. Any measurement error of the external instrument is propagated to the results of the identified parameters. For that reason, an instrument for data acquisition should be, at least, one magnitude order more accurate than the mechanism whose parameters are going to be identified.

A direct geometric transformation can be established, providing the global reference system can be measured by means of an external measurement instrument such as a laser tracker or a coordinate measuring machine. In this way, this transformation obtains the coordinates of the measured points in the global reference system of the mechanism. Thus, direct comparisons in the objective function, between nominal and measured data, can be made.

However, this relation is not usually easy to obtain through a direct measure, and the problem is usually solved by means of least-square methods. These methods allow us to obtain an approximation of this transformation, and this approximation will depend on the configurations used in data acquisition and on the mechanism error in the evaluated points [13].

The home position is a position, within the AACMM working range, where all joint angles have a predefined value. The displacements of the probe are usually measured with respect to this defined position. In [14], Kovac and Frank developed a high precision gauge instrument for the parameter identification procedure and evaluation tests.

The determination of the number of required specific positions is not generalizable from one AACMM to another, since the errors committed by each arm will depend on their configuration and assembly defects. The performance of tests that characterize the influence of each joint on the final error, to finally choose positions in accordance with this influence, must therefore be carried out before defining the capture positions to identify the kinematic parameters. The positions selected for the identification procedure should cover the maximum joint rotation range to cover the influences of all the measuring arm elements in the workspace. In [15], Zheng et al. obtained the spatial error distribution model by using support vector machine theory.

The third step is optimization or geometric parameter identification, and the objective is to search for the optimum values of all parameters included in the model that minimizes the error in the performed measurements. This step is usually carried out by means of approximation procedures based on least-square fitting. This function can be defined as the quadratic difference of the error (obtained between the measured value and the value computed by the kinematic model). The increment established for parameters must be defined for each iteration. In most of the cases, numerical optimization techniques are used to minimize the error. The Levenberg-Marquardt (L-M) [16] method is one of the most widely used techniques to solve the numerical optimization algorithm. This method usually presents lower computational cost, providing a solution closer to the optimum solution for the set of parameters considered. Moreover, the L-M algorithm solves numerical problems that appear in other numerical optimization techniques such as those based on the gradient or on the least-square methods such as the Gauss-Newton. A multiobjective optimization scheme is developed in [17] to solve the nonlinear optimization problem. In [18], Santolaria et al. presented a kinematic parameter estimation technique, which allows us to improve the repeatability of the AACMM by more than 50%. This technique uses a ball bar gauge to perform the data acquisition procedure.

The fourth step, the model evaluation, consists of evaluating the mechanism behavior with the set of the identified parameters obtained in the geometric parameter identification procedure, in configurations different from those used in the optimization process. In [19], Koseki et al. evaluated the accuracy of a mechanism by means of a laser tracking coordinate measuring system.

Finally, an identification of the error sources and a modelling and implementation of the correction models can be optionally performed.

In [20], Piratelli-Filho et al. developed a virtual sphere plate, having a standard deviation of around 0.02 mm and a measurement uncertainty from 0.02 to 1.64 *μ*m in point measurements, to evaluate the measurement performance of AACMM's. The AACMM performance test using the virtual sphere plate resulted in a mean error of 0.023 mm and a standard deviation of 0.039 mm.

A laser tracker is a large-scale measuring instrument with high accuracy. These mechanisms are considered very reliable [21]. Consequently, laser trackers have been used recently instead of other traditional methods such as theodolites or collimators in multiple applications such as robot tracking, testing, calibration, and maintenance. These systems use interferometry for measuring relative distances and optical encoders in order to measure azimuth and elevation angles of a beam-steering mirror. The interferometer measurements are obtained relative to the starting point. Besides, this beam must track the positions of a retroreflector. A plane mirror mounted on a high precision universal joint deflects the beam and hits the retroreflector. This element consists of three perpendicularly oriented plane mirrors, and the beam is reflected parallel. Theoretically, the laser beam hits the center point of the retroreflector. When there is no relative movement between the laser tracker head and the retroreflector, there is no parallel displacement between the emitted and the reflected beam. However, when the retroreflector starts moving, there is a displacement of the reflected laser beam, since, in this case, the laser beam does not hit the center point of the retroreflector. In [22], Lin and Her used a laser tracker system to measure the volumetric errors of a precision machine. The technique presented is based on the ASME B5.54 standards and offers a quick method to characterize these errors. This measuring instrument can also be used in multilateration for verification of machine tools, as presented in [23], where the error order can also be in the error range of a laser tracker. In [21], a laser tracker was used also to analyse the performance of an indoor GPS.

The multilateration technique is a procedure which can be used to improve the mechanism accuracy. This method allows us to reduce the measurement uncertainty by eliminating the angular noise. To achieve this, the multilateration obtains the half point position starting from data of several laser trackers, located in different positions [24]. In [25], Zhang et al. presented specific recommendations for optimization of multilateration set-ups and measurement plans and for minimizing measurement uncertainty. Besides, the authors evaluated the volumetric measurement error propagation, obtaining an average standard deviation of length measurements around 1.2 *μ*m. Hughes et al. [26] presented a laser-interferometric measuring station and obtained the displacement measurement uncertainty, which was used to predict a volumetric uncertainty for a multilateration system, consisting of eight measuring stations and four targets. The combined uncertainty of the measuring station displacement measurements and the CMM repeatability obtained was around 200 nm. In [27], Kim et al. developed a volumetric interferometer system and minimized least-square errors by fitting the measured values to a geometric model of multilateration, obtaining a volumetric uncertainty of less than 1 *μ*m.

The aim of this work is to improve the accuracy of an AACMM by means of a new calibration procedure based on laser tracker multilateration. Although both instruments present the same order of magnitude with respect to the accuracy, the multilateration techniques allow us to capture points with an uncertainty much smaller than the one obtained with an AACMM. The points captured from the multilateration are therefore considered as nominal data in the calibration procedure of the AACMM.

## 2. AACMM Kinematic Model

The AACMM kinematics relates the joint variables and the probe position for any arm posture.

The direct kinematic model is used to calculate the positioning and orientation of the AACMM probe on the basis of certain values of the joint variables, according to the following:

(1) $$\begin{array}{c} y=f\left(\theta_{i},q\right), \end{array}$$

with *i* = 1,…, *n* for an arm with *n* rotating joints. *θ*_*i*_ is given by the vector of the joint variables and *f* represents the model defined, which depends on the parameter vector *q*.

The non-linear equation system to model the mechanism can be developed by applying the Denavit-Hartenberg (D-H) method [6] to every chain in the mechanism. This method has been widely used in mechanism modelling [28, 29] and uses four parameters (distances *d*_*i*_, *a*_*i*_, and angles *θ*_*i*_, *α*_*i*_) to model the coordinate transformation between successive reference systems. The homogenous transformation matrix between frame *i* and *i* − 1 depends on these four parameters:

(2) Aii−1=Tz,d·Rz,θ·Tx,a·Rx,α=[cos⁡θi−cos⁡αi·sinθisinαi·sinθiai·cos⁡θisinθicos⁡αi·cos⁡θi−sinαi·cos⁡θiai·sinθi0sinαicos⁡αidi0001].

The AACMM model used in this work is a Faro Platinum Arm having seven axes, with a nominal value of 2*σ* in the single point articulation and performance test of 0.030 mm according to the specifications of the manufacturer.

**Figure 1:**
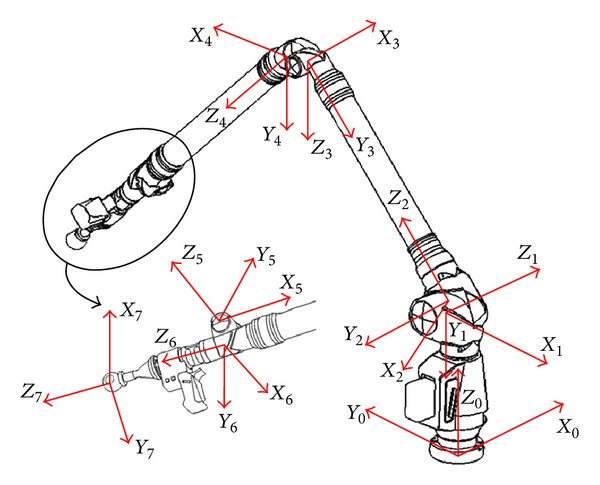
AACMM reference systems.

Figure 1 shows the reference systems used in the AACMM model.

The arm global transformation matrix allows us to express the probe sphere center coordinates with respect to the base of the AACMM. This matrix can be obtained by calculating successive coordinate transformations by premultiplying the transformation matrix between a frame and the previous one, as shown in the following:

(3) [X,Y,Z,1]AACMM′=T60· [X,Y,Z,1]Probe′.

In this equation, 0 is the global reference system of the base and 6 corresponds to the reference frame that moves with the rotation of the last joint.

A reference system is usually defined in the probe. However, the aim of this study is to obtain the sphere center coordinates of the static probe, so this reference system is not necessary. Seven reference systems are used to model the AACMM. The last reference system is located in the center of the reflector and is oriented as reference system six. Thus, the number of parameters for the 6 degrees of freedom (Dof) AACMM is 28. The kinematic model is fully described in [18].  Table 1 shows the initial values for the AACMM D-H parameters.

## 3. Multilateration System

The multilateration technique allows us to reduce the measurement uncertainty by eliminating the angular noise. To achieve this, the multilateration obtains the weighted-point position by means of several laser tracker data, located in different positions. A minimum of three measurements of each point is necessary. Each laser tracker measures the distance from the laser tracker to the target points. These measurements present a noise having a radial component and two angular components. The aim of the multilateration is to decrease the measurement uncertainty, so this technique only uses the radial component of the laser tracker measurements, *m*_*i*_, thus decreasing the measurement noise influence, which allows us to decrease the global uncertainty. These components define a sphere. The intersection of the three spheres obtained by measuring the same point by three laser trackers provides two points. Equations from (4) to (6) provide the spheres obtained by the measurements of each laser tracker, respectively. Consider

(4) $$\begin{array}{c} m_{0}^{2}={\left(x-x_{1}\right)}^{2}+{\left(y-y_{1}\right)}^{2}+{\left(z-z_{1}\right)}^{2}, \end{array}$$

(5) $$\begin{array}{c} m_{1}^{2}={\left(x-x_{2}\right)}^{2}+{\left(y-y_{2}\right)}^{2}+{\left(z-z_{2}\right)}^{2}, \end{array}$$

(6) $$\begin{array}{c} m_{2}^{2}={\left(x-x_{3}\right)}^{2}+{\left(y-y_{3}\right)}^{2}+{\left(z-z_{3}\right)}^{2}. \end{array}$$

The knowledge of the locations of the reference systems of the laser trackers allows us to obtain the following equations:

(7) $$\begin{array}{c} x=\frac{\left(m_{0}^{2}-m_{1}^{2}+x_{2}^{2}\right)}{2x_{1}}, \end{array}$$

(8) $$\begin{array}{c} y=\frac{\left(m_{0}^{2}-m_{2}^{2}+x_{2}^{2}+y_{2}^{2}-2xx_{2}\right)}{2y_{2}}, \end{array}$$

(9) $$\begin{array}{c} z=\pm {\left(m_{0}^{2}-x^{2}-y^{2}\right)}^{1/2}. \end{array}$$

In this work, for simplicity, the unknown reference system location of the three laser trackers has been defined as (0,0, 0), (*x*_1_, 0,0), and (*x*_2_, *y*_2_, 0) with respect to the multilateration reference system.

**Figure 2:**
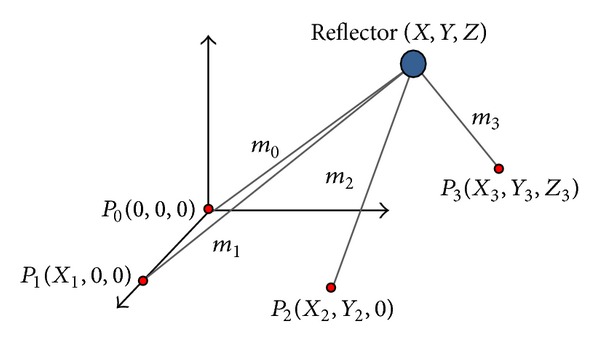
Coordinates of the four laser tracker reference systems expressed in the multilateration system.

A fourth laser tracker can be used to avoid the sign ambiguity in the *Z* coordinate obtained in (9). The reference system of this laser tracker is given by (*x*_3_, *y*_3_, *z*_3_). This point should belong to a plane different from the plane *XY* formed by the other three laser tracker reference systems as shown in Figure 2. In this figure, *P*_*i*_ represents LT_*i*_ position system for the *i* laser trackers. The origin of the multilateration global reference system is given by  *P*_0_.

The (*x*, *y*, *z*) target coordinates are obtained in linear matrix form by operating (7), (8), and (9), as expressed in the following:

(10) [xyz]=−0,5·[1x100−x2x1y21y20−(x3x1z3)+(x2y3x1y2z3)−y3y2z31z3]·[m12−m02−x12m22−m02−x22−y22m32−m02−x32−y32−z32].

The multilateralized coordinates, obtained in (10), will be considered as the nominal coordinates in the identification parameter procedure.

## 4. Data Acquisition

The data acquisition step consists of capturing the nominal coordinates in the workspace of the AACMM. The suitable number of positions is not generalizable from one measuring arm to another, since each measuring arm error will depend on their configuration and assembly defects. The identification procedure should cover the maximum range of joint rotation to consider all the influences of the measuring arm elements.

**Figure 3:**
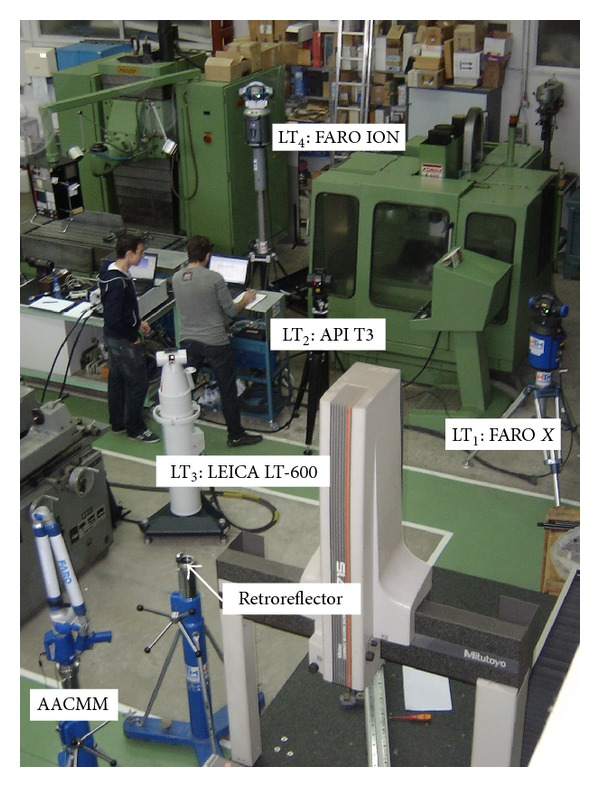
LTs and AACMM distribution used in tests.

In this study, a cloud of points located within the arm workspace was measured simultaneously with both the AACMM (measured values) and four LTs which conform to the multilateralized system (nominal values). The laser tracker FARO *X* model was used as LT_1_, API T3 as LT_2_, LEICA LT-600 as LT_3_, and FARO ION as LT_4_. The positions were distributed throughout the workspace of the arm and reached different arm angle values. The LTs were distributed forming the multilateration global coordinate system, as it was detailed in Section 3. LT_*i*_ positions have been chosen in function of the reflector visibility, thus forming a spatial angle as near as possible to 90° between them. The measurement uncertainty is lower in this position, as demonstrated in a previous work [30]. The AACMM has been arranged in a position that maximizes the visibility of the LTs, as shown in Figure 3.

**Figure 4:**
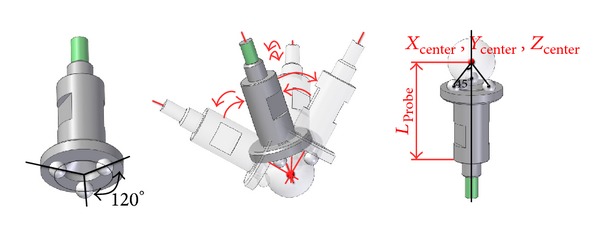
Probe used in the data acquisition procedure.

The AACMM data acquisition technique is usually performed by means of discrete contact probing of surface points of the gauge in order to obtain the center of the spheres from several surface measurements. The time required for the capture of positions is high, and therefore, identification is generally carried out with a relatively low number of arm positions. In this work, a probe presented in [18], capable of directly probing the center of the spheres of the gauge without having to probe surface points, has been used. This probe consists of three tungsten carbide spheres of 6 mm in diameter, laid out at 120° on the probe, as can be seen in Figure 4.

The probe used allows us to define a probe with zero probe sphere radius and with a distance from the position of the housing to the center of the probed reflector sphere of 1.5 inches, allowing direct probing of the sphere center when the three spheres of the probe and the sphere are in contact.

One of the advantages of this type of probe is that the massive capture of arm positions can be performed corresponding to several points of the workspace, which leads to save a considerably amount of time.

23 positions of the retroreflector were measured, from which 21 positions were considered in the parameter identification process (identification positions) and the other 2 positions were kept for the parameter evaluation procedure (test positions). A software developed captured the AACMM measurements, saving the AACMM joint angles, *θ*_*i*_. These angles and the AACMM parameters are the input to the kinematic model. The solution of the non-linear equations by the L-M algorithm obtains the measured point coordinates in the AACMM reference system.

The data acquisition procedure was performed trying to capture data in symmetrical trajectories in the retroreflector to minimize the effect of probing force on the gauge.

**Figure 5:**
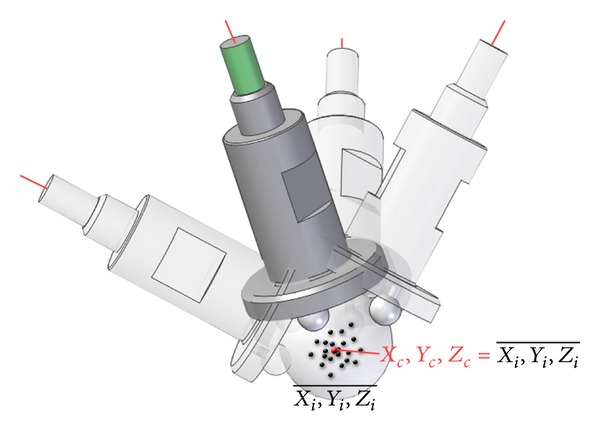
Retroreflector center.

Although the measuring of the retroreflector center with the kinematic mount probe from different arm orientations should result in the same point measured, the unsuitable value of the nominal kinematic parameters of the model will be shown by way of a probing error, resulting in different coordinates for the same measured point in different arm orientations. For that reason, five measurements were taken for each point and the mean point of the set of points captured was considered as the center of the retroreflector measured in the AACMM reference system, as shown in Figure 5.

The distance between the different retroreflector measured positions can be obtained from the points captured by means of the Euclidean distance between each pair of retroreflector positions, obtaining *d*_*ij*_AACMM_, where *i* and *j* represent two measured positions.

At the same time, the four LTs simultaneously measured the distance from the captured point to the local coordinate system.

Diagonal distances are obtained according to the following:

(11) $$\begin{array}{c} r=m+l, \end{array}$$

where *m* is the incremental output of the displacement transducer used and *l* is the offset, which should be calibrated before the multilateration implementation.

The self-calibration and determination of the parameter *l* can be carried out by capturing some additional objective measurements in some different positions. By performing the quadrilateration  *k*  objective point times, the number of equations is given by 4*k* as shown in the following:

(12) (rik)2=(mik+li)2=(xk−xi)2+(yk−yi)2+(zk−zi)2,

for *i* = 0,…, 3 and *k* = 0,…, *k* − 1.

The system unknowns are the four offsets, *l*_*i*_, and the position of the coordinates, *x* ^*k*^,  *y* ^*k*^,  *z* ^*k*^ (3*k* unknowns). To identify the four references, 12 unknowns, corresponding to *x*_*i*_, *y*_*i*_, *z*_*i*_, must be added. Thus, the number of system unknowns is given by 3*k* + 16.

The unknowns can be obtained by the least-square method to minimize the error.

The multilateration technique must solve the objective function defined by

(13) ϕ=∑i=121∑j=14[(xi−xLTj)2+(yi−yLTj)2+(zi−zLTj)2     −(mij+lLTj)2],

where (*x*_*i*_, *y*_*i*_, *z*_*i*_) represents the measured point coordinates in the multilateration reference system, (*x*_LT_*j*__, *y*_LT_*j*__, *z*_LT_*j*__)  is the origin of the LT_*j*_ reference system, *m*_*ij*_ are the measured distances for every point in the LT_*j*_ reference system, and *l*_LT_*j*__ represents the LT_*j*_ offset. The non-linear system obtained by the multilateration technique was solved by means of the L-M algorithm, and the solution obtained gives the following information:

- the point coordinates in the multilateration system: (*x*_*i*_,*y*_*i*_,*z*_*i*_)|_SR  Multi*l*_,
- the laser tracker offsets: *l*_LT_*j*__,
- the origin of the reference systems for the four laser trackers in the multilateration system: (*x*_LT_*j*__,*y*_LT_*j*__,*z*_LT_*j*__)|_SR  Multi*l*_.

The calculated distances between each pair of retroreflector positions can be obtained from the point coordinates in the multilateration system, obtaining *d*_*ij*_Multi*l*_, where *i* and *j* represent the two positions.

## 5. Parameter Identification Procedure

Once the data acquisition technique has been carried out, the parameter identification procedure can be performed.

**Figure 6:**
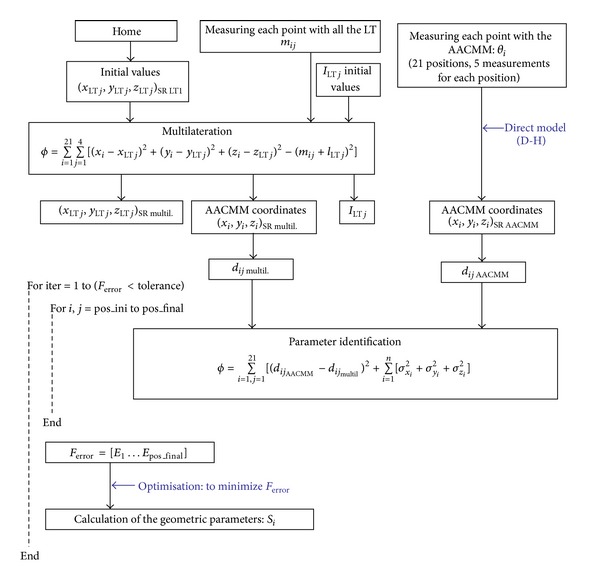
Scheme of the calibration procedure.

Figure 6 shows a scheme of the calibration procedure.

The origin of the laser trackers was measured with respect to the reference system of LT_1_. This reference system has been considered the multilateration reference system for obtaining the initial values of the LT_*j*_ reference systems.

The kinematic parameter identification procedure can be performed starting from the measured and calculated distances (obtained as explained in Section 4). The parameters considered in the AACMM kinematic model are given by (*a*_*i*_, *α*_*i*_, *d*_*i*_, *θ*_0*i*_, *θ*_*i*Enc_), where *θ*_*i*Enc_ are the angles measured by the encoder. The model obtained is a non-linear equation system, and it was solved by the L-M algorithm. The objective function defined in this step considers both the measured and calculated distances, as shown in

(14) ϕ=∑i=1,j=1p[(dijAACMM−dijMultil)2]+∑i=1n[σxi2+σyi2+σzi2],

where *p* represents the number of positions considered in the parameter identification procedure and  (*σ*_*X*_*ij*__, *σ*_*Y*_*ij*__, *σ*_*Z*_*ij*__)  represents the standard deviation of the points measured in each position and each coordinate, showing the influence of the volumetric accuracy and point repeatability.

## 6. Calibration Results

In the optimization process, the distances from each point to every point are taken into account, obtaining 253 distances between the 23 points.

The multilateration procedure described above was carried out based on the 23 captured points for each LT corresponding to the reflector positions used.  Table 2 shows the results obtained. As the initial values for this procedure, null values were assigned for the offsets of all LTs, and for the origin points the corresponding coordinates were those that better fit the distribution observed taken from the measured home points of LT_2_,  LT_3_, and LT_4_ from LT_1_, where the origin of the multilaterized reference system is located.

**Figure 7:**
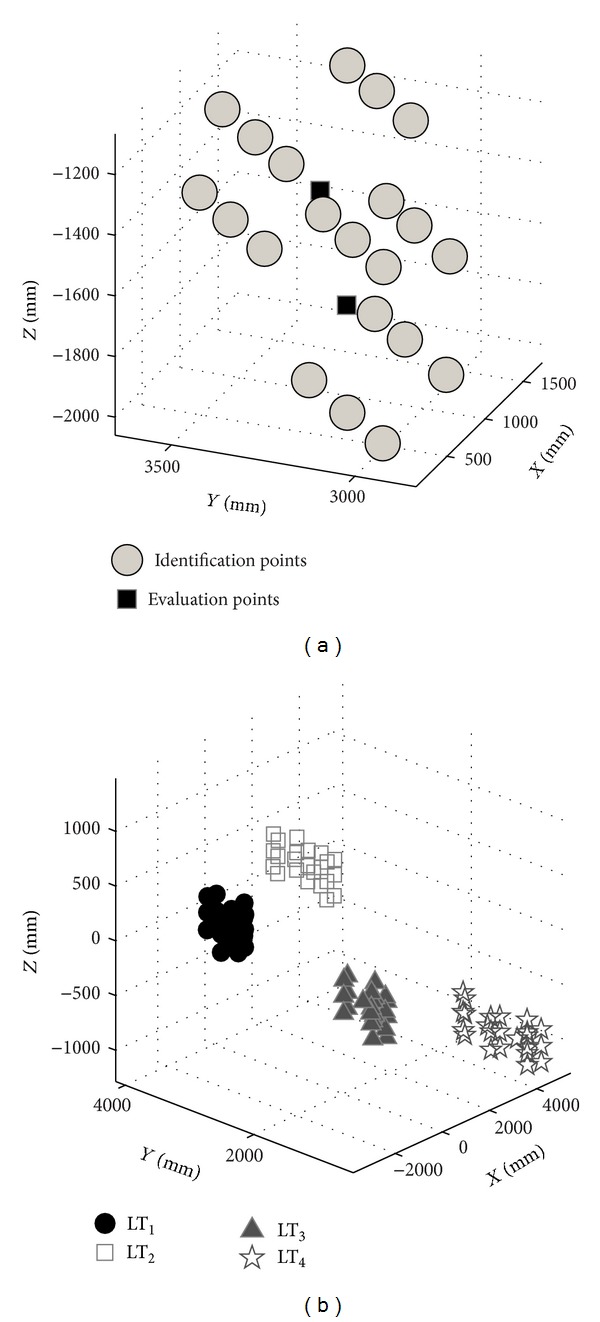
Points captured by LTs. (a) Multilaterated points used as nominal data for the parameter identification procedure; (b) points captured by each LT.

In Figure 7, the coordinates of the captured points by each LT and the coordinates of the multilaterated points, used as nominal coordinates in the parameter identification procedure of the AACMM, are shown graphically.

**Figure 8:**
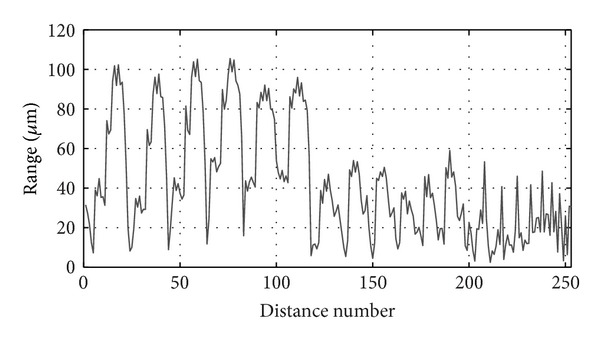
Range of each distance considered in the identification process. Each data represents the difference between the maximum and minimum values of each distance calculated with the measurements made by the four LT's.

As stated before, in order to introduce redundancy in the objective function and thereby restrict to the nominal points the final points obtained with the identified parameters of the measuring arm, all possible distances between the multilaterated points are calculated. This way, we can obtain 253 distances, which will be used as nominal data in the objective function (14) in the identification procedure. In Figure 8, the range of each of the calculated distances in the measurements made by the four LTs can be observed. Thus, each data represents the difference between the maximum and minimum values of each considered distance calculated from the set of the 4 LTs measured data. The distances are arranged starting from position 1 of the retroreflector. The maximum range value obtained by calculating the distances with the 4 LTs was 105 *μ*m, while the mean range for all set of distances was 43 *μ*m.

Following the scheme presented in Figure 6, we can calculate all possible distances between spheres as well as the standard deviations measured with the AACMM from the saved angular data obtained during the data capture process and the set of initial parameters of the AACMM mathematical model shown in Table 1. In accordance with the optimization for the identification scheme presented in Figure 6, the quality indicators for the set of initial parameters of the AACMM model are shown in Table 3. Moreover, Table 3 shows in its first column the distances maximum error obtained for all the reflector points and the index of the points that determine the distance in which the maximum error is calculated.

Analogously, the mean distance errors for all the evaluated distances are also shown. With respect to the standard deviation, Table 3 shows, besides the maximum and mean values, the index of the point and its corresponding coordinate where the greater value is obtained, since in this case the objective function considers the standard deviation for each coordinate independently. As expected, the obtained values are high, considering the initial set of parameters defined for the AACMM mathematical model.

**Figure 9:**
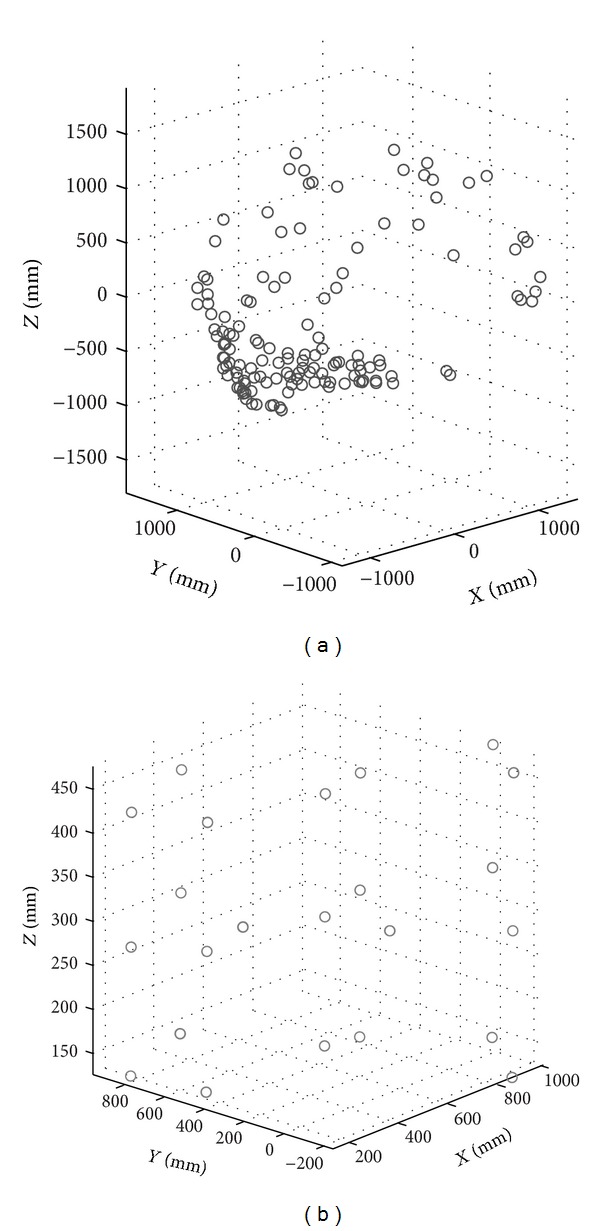
Points captured by the AACMM. (a) Nominal kinematic parameters; (b) identified kinematic parameters.

In the objective function proposed in (14), the data capture setup described for *p* = 21 reflector points, it is necessary to consider the elimination of the terms where *i* = *j* to avoid both the inclusion of null terms and duplicate of distance errors noting that *d*_*ij*_ = *d*_*ji*_. In regard to the standard deviation, each coordinate deviation for every one of the reflector positions is considered. To express mathematically the optimization problem it is required to consider the sum of the calculated quadratic errors. This way, through the objective function in (14), we obtain 231 terms corresponding to the distance errors for the 21 reflector points, plus 63 standard deviation terms of these points, obtaining a total of 294 terms to determine the objective function value in each iteration of the optimization algorithm. This value will contain the influence of the kinematic parameters and the articulation variables considering for this the terms related on the one hand to volumetric precision and on the other to repeatability, for the 105 measuring arm captured positions. In Figure 9, it can be observed graphically the distribution of the captured points in the AACMM reference system, before and after the optimization procedure, while Table 4 shows the identified parameters starting from the initial values of Table 1.

From the identified parameters (Table 4), Table 5 shows the error characteristics results obtained for each of the captured points considering in this case these parameters.

The validation and generalization of the error results calculated for the set of identified parameters over the captured data, to the rest of the AACMM work volume, imply in the more restrictive case obtaining error and deviation values less than the maximum values obtained in this case (Table 5) for any evaluated position of the measuring arm. For this reason, the assessment of the AACMM error in different positions to the ones used in the identification procedure is highly recommended. As shown in Table 5, a maximum error of 118 *μ*m and a mean error of 48 *μ*m for the measuring volume have been obtained, considering the 21 nominal points used in the identification. In normal operation of the measuring arm in this work volume, it is expected to get error values close to the mean value and obtaining maximum error values only in certain arm configurations.

As a last step of the identification procedure, it is necessary to evaluate the set of parameters obtained in different arm positions from those considered in its own identification procedure, such that it is possible to conclude that the error results can be considered reliably within the measuring arm work volume. Furthermore, it is expected that the more similar the measuring arm positions are, when probing the reflector points, to the ones used in the identification, the closer the error results should be to the ones shown in Table 5. Therefore, the points and positions for evaluation must be different from the ones used in the identification procedure. To illustrate this characteristic, in this case two extra positions of the reflector have been considered as test points (Figure 7). For each one of these new reflector positions, 10 angle combinations have been captured corresponding to the center positions of each one of them, captured in the same captured conditions compare to the rest of the points. The nominal distance between these two points, calculated as the Euclidean distance of the multilaterated coordinates, was 492.7164 mm. The distance obtained, using the mean points expressed in the AACMM reference system of the 10 probed points of each point and the identified parameters, was 492.7695 mm, obtaining an error in this case with respect to the nominal of 53 *μ*m, while the maximum standard deviation for the two probed points has been calculated in the *X* coordinate of the first probed point, with a value of 0.1426 mm for all the 10 captured positions. It is therefore possible to conclude that the obtained error value for the identified parameter set can be generalizable to the evaluated work volume. For this reason, the assessment of more evaluation positions different from the one used as an example is recommended when increasing the work volume to be identified. The ideal is to obtain error values always below the identification maximum error, although it is possible to set an acceptable error percentage above the obtained maximum error in order to set a characteristic value of the measuring arm global error according to a less restrictive criterion.

## 7. Conclusions

In this work, a novel calibration technique for parameter kinematic identification of an AACMM is presented. This calibration technique is based on an objective function that considers the volumetric error and repeatability by means of the distance errors and the standard deviation of physical probed points, respectively. Moreover, a new procedure to obtain nominal gauge values for this calibration technique is carried out. This new procedure is based on the measurements of a calibrated spherical retroreflector with 4 LTs. Although the error range of this type of measuring instruments has an order of magnitude similar to the AACMM for this work distances, the use of multilateration techniques can be of great help to reduce the measurement uncertainty, taking as nominal data only the measurements of the LTs. By combining the aforementioned measurements and after the described optimization procedure, is possible to obtain points that can be used as nominal points, in this case materializing distances between them for their use in the AACMM parameter identification procedure. Even though in this work 4 LTs have been used to eliminate the *Z* coordinate sign ambiguity of the multilaterated points, it is possible to realize this procedure using only 3 LTs by making sure that all of the points have the same sign with respect to the multilateration reference system. This way, in the cases when access to this type of measuring instruments is available, it is possible to carry out an AACMM identification procedure without the use of common physical gauges used in this type of procedures.

Finally, the simplification of the calibration procedure presented in this work can be achieved by applying sequential multilarization; thus the use of only one LT is needed to carry out the adapted procedure, with the aim of reducing costs.

## Figures and Tables

**Table 1:** Initial values for the AACMM D-H parameters.

Joint	*θ*_*i*_ (°)	*α*_*i*_ (°)	*a*_*i*_ (mm)	*d*_*i*_ (mm)
1	−90	−90	−40	300
2	135	−90	−40	0
3	180	−90	−34	590
4	−90	90	34	0
5	−90	−90	−34	588
6	180	−90	−34	0
7	0	0	0	318.2

**Table 2:** Multilaterated offsets and origin coordinates obtained in multilateration reference system. 2798 iterations and objective function value below 1 *μ*m.

	Offsets (mm)		Origin coordinates (mm)		
			*x*_LT_	*y*_LT_	*z*_LT_
LT1	*l*_LT1_	1.51695	0	0	0
LT2	*l*_LT2_	0.89275	2262.33138	0	0
LT3	*l*_LT3_	0.18749	1713.00335	2098.93521	0
LT4	*l*_LT4_	2.13351	1913.00558	−343.41959	3010.23578

**Table 3:** Quality indicators for the initial values of model parameters over 21 SMR locations (105 AACMM positions).

Distance error (mm)		2*σ* by retroreflector point (mm)	
Maximum	38.50723	Maximum	22.00413
Causing dist.	4–10	Causing point	17
Medium	17.28859	Causing coord.	X
		Medium	8.17533

**Table 4:** Identified values for the AACMM model parameters by L-M algorithm.

Joint	*θ*_*i*_ (°)	*α*_*i*_ (°)	*a*_*i*_ (mm)	*d*_*i*_ (mm)
1	−89.1269	−89.9216	−42.4313	300
2	134.7734	−89.7178	−41.7885	1.2337
3	184.2704	−90.1202	−28.9084	591.2423
4	−95.4182	89.5364	29.4449	0.6300
5	−88.0773	−89.9482	−28.5745	591.5284
6	181.0832	92.5750	27.5479	12.2143
7	0.2117	0	0.4124	254.0575

**Table 5:** Quality indicators for the identified values of model parameters over 21 SMR locations (105 AACMM positions).

Distance error (mm)		2*σ* by retroreflector point (mm)	
Maximum	0.11824	Maximum	0.20644
Causing dist.	2–12	Causing point	4
Medium	0.04825	Causing coord.	Y
		Medium	0.11545
